# Genomic Sequencing and Comparison of Sacbrood Viruses from *Apis cerana* and *Apis mellifera* in Taiwan

**DOI:** 10.3390/pathogens10010014

**Published:** 2020-12-28

**Authors:** Ju-Chun Chang, Zih-Ting Chang, Chong-Yu Ko, Yue-Wen Chen, Yu-Shin Nai

**Affiliations:** 1 Department of Biotechnology and Animal Science, National Ilan University, Yilan 260, Taiwan; c1887g@gmail.com (J.-C.C.); a0923653853@gmail.com (Z.-T.C.); ricardo7677@gmail.com (C.-Y.K.); 2 Department of Entomology, National Chung-Hsing University, Taichung 402, Taiwan

**Keywords:** sacbrood virus, sacbrood disease, *Apis cerana*, *Apis mellifera*

## Abstract

Sacbrood virus (SBV) was the first identified bee virus and shown to cause serious epizootic infections in the population of *Apis cerana* in Taiwan in 2015. Herein, the whole genome sequences of SBVs in *A. cerana* and *A. mellifera* were decoded and designated AcSBV-TW and AmSBV-TW, respectively. The whole genomes of AcSBV-TW and AmSBV-TW were 8776 and 8885 bp, respectively, and shared 90% identity. Each viral genome encoded a polyprotein, which consisted of 2841 aa in AcSBV-TW and 2859 aa in AmSBV-TW, and these sequences shared 95% identity. Compared to 54 other SBVs, the structural protein and protease regions showed high variation, while the helicase was the most highly conserved region among SBVs. Moreover, a 17-amino-acid deletion was found in viral protein 1 (VP1) region of AcSBV-TW compared to AmSBV-TW. The phylogenetic analysis based on the polyprotein sequences and partial VP1 region indicated that AcSBV-TW was grouped into the SBV clade with the AC-genotype (17-aa deletion) and was closely related to AmSBV-SDLY and CSBV-FZ, while AmSBV-TW was grouped into the AM-genotype clade but branched independently from other AmSBVs, indicating that the divergent genomic characteristics of AmSBV-TW might be a consequence of geographic distance driving evolution, and AcSBV-TW was closely related to CSBV-FZ, which originated from China. This 17-amino-acid deletion could be found in either AcSBV or AmSBV in Taiwan, indicating cross-infection between the two viruses. Our data revealed geographic and host specificities between SBVs. The amino acid difference in the VP1 region might serve as a molecular marker for describing SBV cross-infection.

## 1. Introduction

Sacbrood virus (SBV) is a single-stranded, positive-sense RNA virus that belongs to the *Iflaviridae* family [1,2,3]. The particles of SBV are 28 nm in diameter, nonenveloped, icosahedral [4,5]. SBV is a common honeybee virus that exhibits a high prevalence of infection mainly in early larval stage of honeybees. This condition affects the broods of honeybees, and the specific symptoms can be easily identified in dead deformed larvae in hives with fluid-filled sacs [6,7,8,9].

Sacbrood disease, which is caused by SBV infection, was first reported and verified in *Apis mellifera* in 1964 [7,10]. The ectoparasitic mite *V. destructor* plays a role in SBV transmission [11]. Infection with SBV (AmSBV) is now commonly found in *A. mellifera* worldwide and does not usually result in *A. mellifera* colony loss [12,13,14,15,16]. However, according to a previous report from 1976, the SBV found in *A. cerana* (AcSBV) has large impacts on *A. cerana* in several Asian countries, including China, Korea, India, Vietnam, and Thailand [17,18,19,20,21,22]. During 1991–1992, an outbreak of sacbrood disease caused up to 90% colony losses in Thailand [23,24,25,26,27,28,29,30]. As mentioned above, AcSBV infection usually causes a high rate of *A. cerana* larvae death and may even lead to whole-colony collapse [31,32].

The *A. cerana* is an indigenous honeybee species in Taiwan. The natural fitness of *A. cerana* is better than *A. mellifera*. The *A. cerana* has a higher tolerance for low temperature and better performance on the pollination in the mountain regions than *A. mellifera*. Therefore, *A. cerana* contributes to the pollination of mountainous orchards, including plums and peaches, etc., which are counted for 1.3% of Taiwan agricultural production [33]. Since AcSBV was first detected in Taiwan in 2015, many beekeepers in Taiwan have reported significant *A. cerana* larval death with symptoms caused by AcSBV in *A. cerana* colonies [34,35]. The virus was found to have spread from southern Taiwan to northern and then eastern Taiwan in 2016. Based on long-term surveillance data, the prevalence rate of AcSBV in *A. cerana* colonies had dramatically increased from 47% to ~70% at the end of 2016 and continued to increase to 72% in 2017 [35]. More than 90% of *A. cerana* colonies were influenced by AcSBV infection from 2016–2019, and the colony collapse does have impacts on the sale market of *A. cerana* colonies, price of honey, and the productions of mountainous orchards. The prevalence of SBV in the population of *A. cerana* in Taiwan is now irreversible.

To better understand the relationship between each SBV strain among Asian countries, the analysis of genome sequences from different geographic areas could provide an accurate and reliable method of detecting variations within the same type of genome based on molecular comparisons. Several studies have examined the whole genome sequences of either AmSBV or AcSBV worldwide. In Korea, six AmSBVs (AmSBV-Kor1 [KP296800.1], AmSBV-Kor2 [KP296801.1], AcSBV-Kor3 [KP296802.1], AcSBV-Kor4 [KP296803.1], AmSBV-Kor19 [JQ390592.1], and AmSBV-Kor21 [JQ390591.1]) from *A. mellifera* were sequenced and further compared (Table 1) [24,36]. In 2017, a comparative genomic analysis among nine SBVs of *A. cerana* and *A. mellifera* was performed in Vietnam [30]. These reports identified different genomic features and revealed the genetic diversity among these SBVs, suggesting that viral cross-infections might occur between AcSBV and AmSBV.

According to our previous data, cross-infection might occur between AcSBV and AmSBV in Taiwan [33,35]. However, the available information on the whole genome sequences of AcSBV and AmSBV in Taiwan is insufficient. Therefore, this study attempted to determine and analyze two complete genome sequences of AcSBV and AmSBV in Taiwan. This is the first complete genome sequences of AcSBV and AmSBV from Taiwan. Phylogenetic analysis based on conserved viral proteins and similarity comparisons of the genomic sequences with those of 54 other SBV strains worldwide were also performed, these results may contribute to better understanding the variation of other SBV strains.

## 2. Results

### 2.1. Genomic Sequences and Analysis of SBV Strains in Taiwan

**Figure 1:**
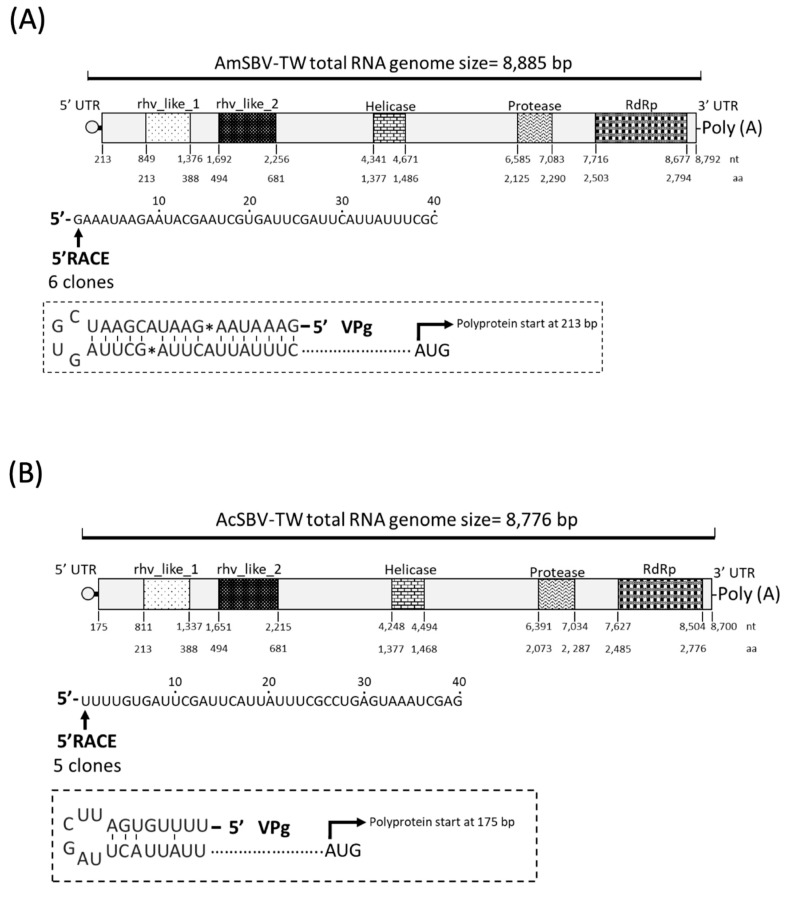
The genomic maps of (**A**) AmSBV-TW (accession number: MN082651) and (**B**) AcSBV-TW (accession number: MN082652). The full-length sequences were obtained using a combination of RT-PCR amplification and rapid amplification of 5′ and 3′ cDNA ends (5′ RACE and 3′ RACE). The nucleotide (nt) and amino acid (aa) positions of each domain was indicated below the schematic of AmSBV-TW and AcSBV-TW, respectively. The 5′ terminal sequences of AmSBV-TW and AcSBV-TW were determined by 5′ RACE, and the prediction of the 5′ secondary structure of AmSBV-TW and AcSBV-TW was performed on the RNAfold WebServer (http://rna.tbi.univie.ac.at/cgi-bin/RNAWebSuite/RNAfold.cgi) and presented in the dotted box. VPg = viral protein genomic linked. * Mismatch nucleotide base.

The whole genomes of AmSBV and AcSBV from *A. mellifera* and *A. cerana* in Taiwan were sequenced. The complete genome sequences of the two SBV strains were deposited in GenBank under the accession numbers MN082651 for AmSBV-TW and MN082652 for AcSBV-TW. The genomes of AmSBV-TW and AcSBV-TW were annotated by using NCBI ORF finder, and the numbers of RNAs encoded by AmSBV-TW and AcSBV-TW were 8885 and 8776, respectively. The 5′ and 3′ untranslated regions (UTRs) of AmSBV-TW were 212 and 115 nt, respectively; for AcSBV-TW, the 5′ and 3′ UTRs were 174 and 272 nt, respectively. Only one open reading frame (ORF) was predicted in the genomic RNA sequence of AmSBV-TW, which extended from nt 213 to 8792, encoding a putative polyprotein of 2859 amino acids. The genomic RNA of AcSBV-TW also encoded one ORF, from nt 175 to nt 8700, encoding a putative polyprotein of 2841 amino acids (Figure 1). Two structural domains were identified as rhv-like domains in the 5′ region of AmSBV-TW and AcSBV-TW, and three nonstructural domains, including *helicase*, *protease,* and *RNA-dependent RNA polymerase* (*RdRp*), were located at the 3′ regions of both AmSBV-TW and AcSBV-TW. The analysis of the protein domain arrangement and genomic structures of AmSBV-TW and AcSBV-TW revealed characteristics of family *Iflaviridae* (Figure 1).

### 2.2. Comparisons of SBV Strains

The sequences of AmSBV-TW and AcSBV-TW were first compared to each other (Table 2). The results of nt sequence comparisons showed that the full-length genomic RNA and ORF regions were highly conserved between AmSBV-TW and AcSBV-TW, sharing 90% identity, while the 5′ and 3′ UTRs showed high variation between AmSBV-TW and AcSBV-TW, presenting 68% and 73% identity, respectively (Table 2). In the amino acid sequence comparisons, the helicase protein domain exhibited the highest identity (99%) and was the most conserved protein domain between AmSBV-TW and AcSBV-TW, followed by rhv_like_2 (97%), RdRp (96%), polyprotein, and rhv_like_1 (95%), while the nonstructural protein protease showed low identity (75%) between AmSBV-TW and AcSBV-TW (Table 2).

The genomic regions of AmSBV-TW and AcSBV-TW were further compared to those of SBV strains from other countries (Table 3). The nucleotide sequences of the whole AmSBV-TW and AcSBV-TW genomes shared identities of 87% (AcSBV-India-II10) to 92% (AcSBV-Viet-SBM2) and 88% (South Australia_1, 2, 3, SBV_MR, MD1, 2 and AcSBV-India-II10) to 96% (AcSBV-Viet1, 2, AmCSBV-SDLY and CSBV-FZ), respectively, with other SBVs (Table 1; Table 3). The identities of the 5′ and 3′ UTRs showed high variation among SBVs; for AmSBV-TW, the 5′ and 3′ UTRs shared 30% (CSBV-SXnor1) to 78% (Korean strain) and 10% (AcSBV-India-K5B) to 94% (MD1 strain) identities, respectively, with those of other SBVs, while AcSBV-TW showed 42% (CSBV-SXnor1) to 93% (AcSBV-Viet3) identity for the 5′ UTR and 11% (AcSBV-India-K5B) to 85% (NT strain) identity for the 3′UTR (Table 1; Table 3).

The amino acid identities among the SBV strains were similar to the variations in the nucleotide identities. In the comparison of polyprotein amino acid sequences, AmSBV-TW was most similar to SBV-UK, with 98% aa identity, and AcSBV-TW was most similar to AcSBV-Viet1 and 2, sharing 98% aa identity. (Table 4). Among the structural proteins (rhv_like_1 and rhv_like_2), AmSBV-TW shared 81% (AmSBV-Viet6) to 98% (NT strain) and 94% (AcSBV-Viet-NA) to 99% (AmSBV-Kor1) identities with those of other SBVs, and AcSBV-TW shared 81% (AmSBV-Viet6) to 98% (CSBV-FZ) and 94% (AcSBV-Viet-BP) to 100% (AmCSBV-SDLY, CSBV-JLCBS-2014 and AcSBV-Viet-BG) identities with those of other SBVs (Table 4). The identities of the nonstructural proteins, including helicase, protease and RdRp, between AmSBV-TW and other SBVs showed greater variation than those of the structural proteins, ranging from 60–100%, 70–98%, and 88–98%, respectively, while the corresponding values were 60–100%, 67–99%, and 89–98% for AcSBV-TW (Table 4).

**Figure 2:**
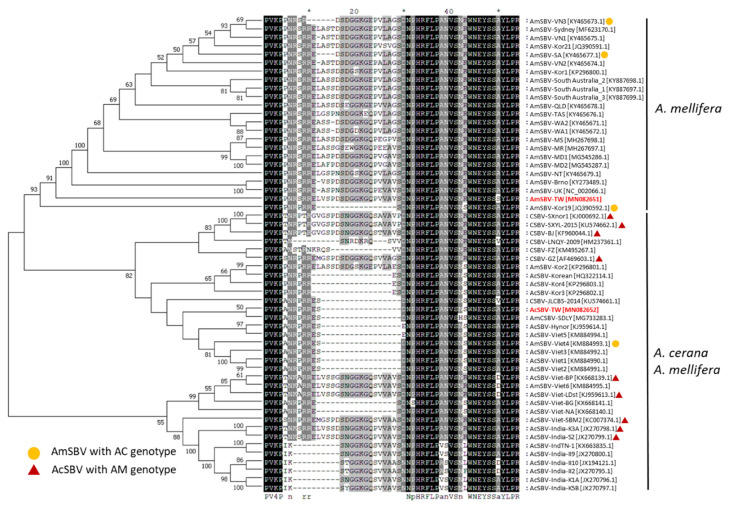
Phylogenetic tree constructed based on the polyprotein amino acid sequences of AmSBV-TW and AcSBV-TW and 54 other SBV strains from the NCBI database. The phylogenetic tree was constructed using the neighbor-joining (NJ) method and 1000 bootstrap replications. The pairwise alignment indicated the deletion patterns in the VP1 region of SBV strains. The round shape symbols indicated AmSBVs with AC genotype (deletion in VP1 region), and the red triangle shape symbols indicated AcSBVs with AM genotype (non-deletion in the VP1 region). red font: The Taiwan strains from this study. *: A note for every 10 bases.

Comparisons of nucleotide and amino acid sequences revealed the deletion of 51 base pairs (17 amino acids) in AcSBV-TW (from amino acid positions 712–730 (VP1 region) in the ORF region) compared to AmSBV-TW, and the same deletion (AC-genotype) was found in most of the other SBVs from *A. cerana*, including AcSBV-Viet1, 2, 3, 5, AcSBV-Hynor, AmCSBV-SDLY, CSBV-JLCBS-2014, AcSBV-Korean, AcSBV-Kor3, 4, AcSBV-Viet-NA, AcSBV-Viet-BG, except AmSBV-Kor19 and AmSBV-Viet4 (Figure 2). Another 10–13-amino-acid deletion was found in six SBVs from *A. cerana* in India and two SBVs from *A. cerana* in China, including AcSBV-India-II2, -II9, -II10, -K1A, -K5B, -TN-1, CSBV-LNQY-2009, and CSBV-FZ (Figure 2). However, a less than 10-amino-acid deletion was found in the SBVs from *A. mellifera* in Australia, including AmSBV-VN3 and SA (Figure 2). Similar to other SBVs from *A. mellifera,* AmSBV-TW showed no deletion in the 712–730 amino acid region of the ORF (AM-genotype), and same to the AcSBVs from China (CSBV-GZ, -BJ, -SXnor1, and SXYL-2015), India (AcSBV-India-K3A and -S2), and Vietnam (AcSBV-Viet-BP, -LDst, and -SBM2) also lacked the 17-amino-acid deletion (Figure 2).

### 2.3. Phylogenetic Analysis

Phylogenetic analysis was performed based on the polyprotein sequences of 56 strains of SBV. The phylogenetic tree clearly diverged into two main branches according to the host (Figure 2). The first branch was composed of the SBV strains from *A. mellifera*; within this branch, AmSBV-TW was closely related to AmSBV-UK and AmSBV-Kor19. The second branch was composed of SBV strains from either *A. cerana* or *A. mellifera*; moreover, a branch contained two groups, one composed of AcSBV from India, while the other consisted of AcSBV and AmSBV from Asian areas, which included AcSBV-TW from Taiwan, CSBV strains from China, AmSBV/AcSBV from Korea, and AmSBV/AcSBV from Vietnam. Especially according to the phylogenetic tree, AcSBV-TW is closely related to AmCSBV-SDLY and CSBV-FZ (Figure 2).

### 2.4. Variation of VP1 Region in AcSBV and AmSBV in Taiwan

**Figure 3:**
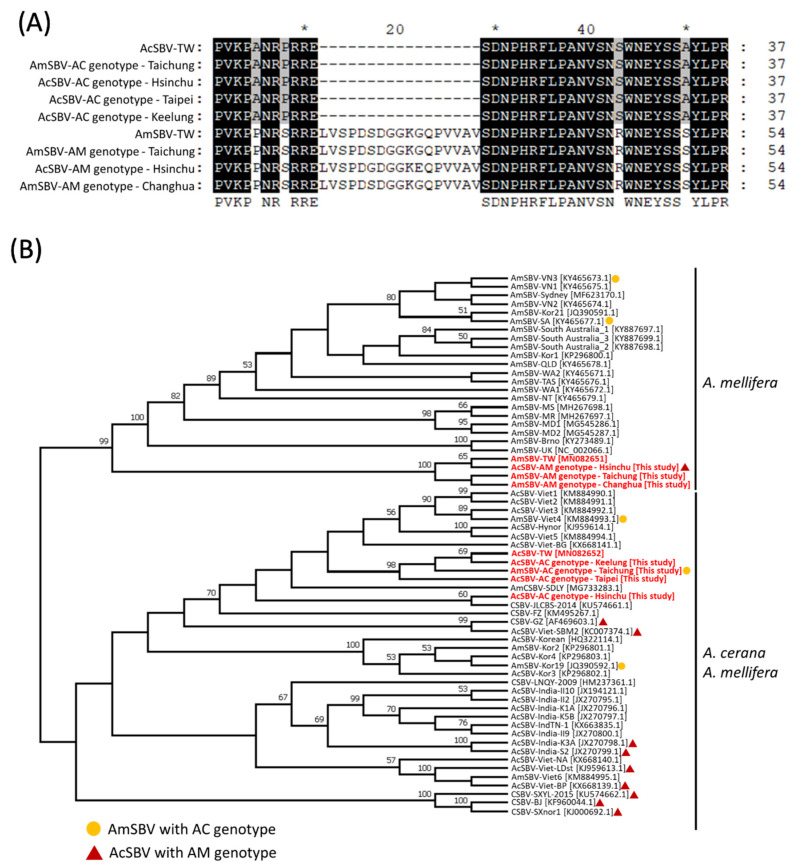
Comparison and phylogenetic analysis of partial VP1 region of AcSBV and AmSBV from Taiwan. (**A**) The pairwise alignment indicated the 17-anmion deletion presented not in AcSBV but also AmSBV vice versa. (**B**) Phylogenetic tree construct based on the partial VP-1 amino acid sequences of Taiwan AmSBV and AcSBV, and other 54 SBV strains from NCBI. The phylogenetic tree was constructed using the neighbor-joining (NJ) method and 1000 bootstrap replications. red font: The Taiwan sequences from this study. *: A note for every 10 bases.

As aforementioned, the deletion of 51 base pairs (17 amino acids) in the VP1 region were found in most of AcSBV and, thereby, named as AC-genotype and vice versa (AM-genotype without any deletion in the VP1 region). To better understand whether the AcSBV AM-genotype and AmSBV AC-genotype exist in the populations of *A. cerana* and *A. mellifera,* the partial VP1 sequence of three AmSBV and four AcSBV from Taiwan were further compared to those of AmSBV-TWand AcSBV-TW (Figure 3A). The results showed that the 17-amino-acid deletion (AC genotype) was only detected in one AmSBV sample in Taichung; besides, one AcSBV with AM genotype was also detected in the sample from Hsinchu (Figure 3A) The phylogenetic analysis was also performed based on the partial VP1 region of 63 strains of SBV. It revealed similar result to those of polyprotein phylogeny. Moreover, the AcSBV-AC genotype and AmSBV-AC genotype in Taiwan were grouped in the same clade, which was closed to CSBV-FZ and CSBV-JL, and the AmSBV-AM genotype and AcSBV-AM genotype in Taiwan were grouped in the same clade, which was closed to AmSBV-UK (Figure 3B). These results supported that the cross-infection between AcSBV and AmSBV in *A. cerana* and *A. mellifera.*

## 3. Discussion

AcSBV has recently been recorded in Taiwan and caused serious losses of *A. cerana* from 2015 to 2019 [33,34,35]. In Taiwan, most *A. cerana* populations are reared in Northern Taiwan, and according to our observations, some of these apiaries are crossbreeding with *A. mellifera* populations. The detection of AcSBV prevalence in *A. mellifera* populations from the sampling sites where *A. cerana* and *A. mellifera* were crossbreeding confirmed that AcSBV prevalence rates gradually developed a similar trend in the *A. cerana* and *A. mellifera* crossbreeding apiaries, and the existence of AcSBV cross-infection between *A. cerana* and *A. mellifera* was also confirmed by phylogenetic analysis based on partial VP1 sequences [33]. Similar to our case, some SBV strains from *A. mellifera* included in this study were found to be distinct from other AmSBV strains in terms of genomic features and were clustered with AcSBVs based on whole genome comparisons and phylogenetic analysis [24,30,36,40]. Strains from the same or closer geography distance showed higher similarity, and the phylogenetic analysis also indicated the same result [24,30,36,40,44]. Indeed, it was shown that the cross-infection of SBV strains occurs between two honeybee species in other countries, including China, Vietnam, and Korea, leading to the high genetic divergence among SBV strains [24,30,36,40].

As mentioned above, the comparison of different genome sequences could provide precise and reliable information for detecting variations within closely related species. In this study, complete SBV genome sequences from *A. cerana* and *A. mellifera* in Taiwan were determined and were designated AcSBV-TW and AmSBV-TW, respectively. Our comparisons revealed greater divergence in 5′ and 3′ UTRs than in ORF region not only between AmSBV-TW and AcSBV-TW but also compared with those of SBVs from other countries (Table 3). The structures of 5′ UTR play many functions in RNA viruses, including viral replication, translation, virus–host protein interactions, and virulence [45,46]. It has been also reported that the 5′ UTR of RNA viruses in *Iflaviridae* functions as an internal ribosome entry site (IRES) [47]. Similar result was also described from [30], that 5′ UTRs of VN-SBVs (including AcSBV and AmSBV in Vietnam) showed greater divergence from SBV strains from other countries [30]. The structure of 5′UTR of SBV might have a critical function for virus replication, therefore the sequence divergence may reveal different viral activities among different viruses.

It should be noted that deletions in the 712–730 amino acid (VP1) region of the ORF were found in most AcSBVs [24,30,40]. Since it has been mentioned that VP1 has the highest sequence variation among SBVs [24,30,40,48]. Based on our comparisons, there are three types of deletion patterns: 17-amino-acid deletions, 10–13-amino-acid deletions, and deletions of less than 10 amino acids (Figure 2). Most of the examined AcSBVs, including AcSBV-TW, exhibit a 17- or 10–13-amino-acid deletion in VP1 region, while there were nine AcSBVs from China, India, and Vietnam exhibiting no deletions, and a deletion of less than 10 amino acids was found in SBVs from *A. mellifera* in Australia, including AmSBV-VN3 and SA. AcSBVs from India all have 10-amino-acid deletion, which were clustered in same branch, suggesting that the occurrence of the 17-amino-acid difference in the VP1 region tends to be host-preference. Interestingly, some AmSBV from Asia countries, where have *A. cerana* population, including AmSBV-Viet4 and AmSBV-Kor19, also harbor the same 17-amino-acid deletion in their VP1 region, indicating the cross-infection of SBV at different geographic origins [49].

Further investigation of the VP1 variations of AcSBV and AmSBV in Taiwan indicated that most AcSBVs have 17-amino-acid deletion in their VP1 region compared to AmSBV, while the AmSBV-AC genotype and AcSBV-AM genotype were also detectable in AmSBV and AcSBV, respectively. It has been reported that high variability exists among SBV genomes, especially between AC-genotype SBV and AM-genotype SBV, and this genetic diversity is supported by the geographic distances or viral cross-infections between different honeybee species [30,40]. These characteristics might also provide clues regarding SBV adaption in different hosts [24,30]. In conclusion, the genomic differences in AmSBV-TW and AcSBV-TW compared with other SBVs could be further applied to identify genetic markers for host-specific and geographic distance evaluations.

The phylogenetic analysis based on the polyproteins and partial VP1 region of AmSBV-TW and AcSBV-TW and other SBV strains revealed that the SBV strains diverged into two distinct branches, which could represent host affiliation and geographic origin. According to comparisons with the current 54 strains of SBV available in NCBI, AmSBV-TW and AcSBV-TW were grouped onto different branches. AcSBV-TW is closely related to AmCSBV-SDLY and CSBV-FZ and was clustered into the AcSBV group with the AC genotype; therefore, it was assumed that the AcSBV in Taiwan may have originated from China and currently be experiencing host adaption and evolution. In contrast, AmSBV-TW was grouped into the AM-genotype SBVs, which originated from *A. mellifera*; however, AmSBV-TW was separated from other AmSBVs in this group, suggesting that geographic distance might be involved in the process of genomic divergence.

The comparison and phylogenetic analysis of partial VP1 region in another seven SBVs in Taiwan showed that most of AcSBV and AmSBV were grouped into AcSBV-TW and AmSBV-TW, respectively, except one AmSBV-AC genotype (grouped with the AcSBV-TW) and one AcSBV-AM genotype (grouped with the AmSBV-TW). These results suggested that AcSBVs in Taiwan presented the closely geographical relationship to those of China, while AmSBVs in Taiwan revealed the geographic distance-based evolution. Additionally, the AcSBV-AM genotype and AmSBV-AC genotype clearly showed the viral cross-infection between these two species. 

## 4. Materials and Methods 

### 4.1. Sample Collection

**Figure 4:**
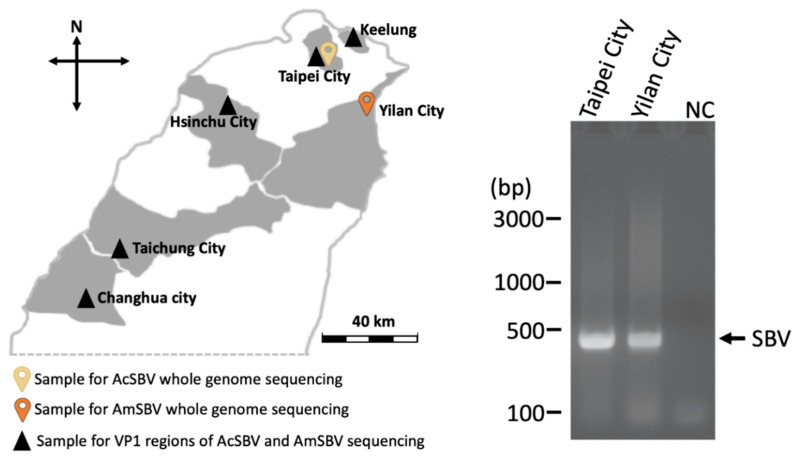
Locations of sample collection and the electrophoretic screening of SBV infection-positive samples by RT-PCR with primer set of VP1-F/VP1-R (Supplementary Table S2). The two sample sites for genomic sequencing were located at northern Taiwan; the AcSBV and AmSBV samples were collected in Taipei City and Yilan City, respectively. The black triangle represents the sampling site for detection of partial VP1 region (335 bp) variations in AcSBV and AmSBV in Taiwan. bp = base pair; NC = negative control. The black arrow indicated the signals of SBV positive.

For vial genomic sequencing, *A. cerana* and *A. mellifera* were collected from two apiaries located in Taipei City and Yilan City, respectively, in 2018 (Figure 4; Supplementary Table S1). Besides, 3 samples of *A. mellifera* and 4 samples of *A. cerana* were selected for the investigation of variations in VP1 region (Figure 4; Supplementary Table S1). The midguts of 10 randomly selected adult bees were collected as a single sample in each apiary. The collected samples were preserved in 0.5 mL of RNA Keeper^TM^ Tissue Sample Storage Reagent (Protech, Taipei, Taiwan) in a 1.5 mL microtube and stored at −20 °C for the following experiment of RNA extraction. 

### 4.2. RNA Extraction and RT-PCR Screening

Each sample was homogenized with a sterile plastic pestle. Total RNA was extracted from the midgut tissues using TRIzol reagent (Invitrogen, Waltham, MA, USA) following the manufacturer’s instructions. The quantity and purity of the RNA were measured using a ScanDrop^2^ Nanovolume spectrophotometer (Analytik Jena, Jena, Germany). For copy DNA (cDNA) synthesis, total RNA (1 μg) samples were treated with DNase I (Roche Molecular Biochemicals, Basel, Switzerland) and then primed with random hexamer primers and reverse-transcribed with Super Script III (Invitrogen, Waltham, MA, USA) at 42 °C for 3 h, after which the reaction was stopped at 70 °C.

All of the samples were first screened with the VP1-F/VP1-R specific primer set (Supplementary Table S2) via PCR with cycling at 95 °C initial denaturation for 45 s and then followed by 35 cycles of 95 °C denaturation for 45 s, 50 °C primer annealing for 45 s, and 72 °C extension for 1 min, followed by a 10 min final extension at 72 °C and storage at 20 °C. The RT-PCR products were analyzed by electrophoresis on a 2% agarose gel in 1× TAE buffer to check the SBV infection-positive samples for the following experiments. For the investigation of variations in VP1 region, the infection-positive samples were amplified by using VP1-F/SBV_R4 primer set (Supplementary Table S2), and the PCR products were subjected to commercial DNA sequencing. 

### 4.3. Whole Genome Sequencing and Assembly of AcSBV and AmSBV

The AcSBV infection-positive samples from *A. cerana* in Taipei City and the AmSBV infection-positive samples from *A. mellifera* in Yilan City were subjected to whole-genome sequencing by RT-PCR with 15 primer sets (Supplementary Table S2). PCR amplification was performed as described above. The RT-PCR products were analyzed by electrophoresis on a 2% agarose gel in 1× TAE buffer [35]. The PCR products with positive signals were purified (Geneaid, New Taipei City, Taiwan) and subjected to commercial DNA sequencing. The obtained sequences were subjected to the genome assembly using SeqMan (DNASTAR, Madison, WI, USA). 

### 4.4. Viral Genomic 5′ and 3′ End Sequencing

The 5′ and 3′ untranslated regions of the AcSBV and AmSBV genomes were obtained by the rapid amplification of cDNA ends (RACE) method, which was slightly modified from [50]. For the 3′ end of the viral genome, 1 µL of an anchor-dTv primer at 50 µM was used to prime 1 µg of total RNA in a 20 µL reaction containing 10 mM dNTPs at 70 °C for 5 min, after which the reaction mixture was placed on ice for 1 min. RNA was reverse transcribed by using Super-Script III (Invitrogen, Waltham, MA, USA) at 42 °C for 1 h, and the reaction was stopped by heating at 70 °C for 15 min. The viral 3′ end sequences were amplified with genome-specific forward primers (GSP-F) and an anchor primer (Supplementary Table S2) using PCR Master Mix (Thermal, Riverside County, CA, USA).

The sequence of the viral 5′ end was decoded as described by [51], with slight modifications [51]. A total of 5 µg RNA was used for 5′ RACE, and the RN was primed with 0.5 µL of the GSP-RT primer (100 ng/µL) in a 20 µL reaction at 80 °C for 3 min, after which the mixture was rapidly transferred to ice. The iScript™ cDNA Synthesis Kit (BIO-RAD, Hercules, CA, USA) was used for reverse transcription at 42 °C for 1 h, and the reaction was inactivated at 95 °C for 5 min. Then, the RNA templates of the cDNA samples were digested with 1.5 U of RNase H (Thermo Fisher Scientific, Waltham, MA, USA) at 37 °C for 20 min. The sample was subsequently cleaned using a GenepHlow™ Gel/PCR Kit (Geneaid, New Taipei City, Taiwan). The 15 µL eluted cDNA sample was treated with transferase (Tdt) in the following reaction mixture: 5 µL of 10 × terminal deoxynucleotidyl transferase (Tdt) buffer (NEB, Ipswich, MA, USA), 5 µL CoCl_2_ (2.5 mM), 0.5 µL dATP (10 mM), and 0.5 µL Tdt for 5′ end tailing, performed at 37 °C for 25 min, and the reaction was then stopped by heating at 70 °C for 10 min. The sample was next subjected to two rounds of PCR amplification using PCR Master Mix (Thermal, Riverside County, CA, USA). For the first round of PCR, 1 µL of cDNA template was used for amplification by three primers: GSP-R1, QO, and QT, at 25 pmols each (Supplementary Table S2). The PCR program was as follows: 98 °C initial denaturation for 5 min, 48 °C annealing for 2 min, 72 °C extension for 40 min, followed by 30 cycles of 94 °C denaturation for 10 s, 50 °C primer annealing for 30 s, 72 °C extension for 2 min, and a final extension at 72 °C for 15 min. The first-round PCR product was diluted 20-fold in ddH_2_O for the second round of amplification. A total of 1 µL of the diluted PCR product and 25 pmols of each of the GSP-R2 and QI primers (Supplementary Table S2) were mixed for PCR amplification via the following program: 98 °C for 5 min, 30 cycles at 94 °C for 10 s, 50 °C for 30 s, 72 °C for 1 min, and a final extension at 72 °C for 15 min. The amplified PCR products were checked by electrophoresis on a 4% agarose gel in 1× TAE buffer. The amplified DNA fragments were purified using a GenepHlow™ Gel/PCR Kit (Geneaid, New Taipei City, Taiwan) and cloned into the TA vector (RBC Bioscience, New Taipei City, Taiwan); the ligated plasmid DNAs were transformed into *Escherichia coli* DH5α (RBC Bioscience, New Taipei City, Taiwan) following the user manual. The plasmids were extracted from cultured bacterial colonies with a Presto™ Mini Plasmid Kit (Geneaid, New Taipei City, Taiwan) and were sequenced bidirectionally with the M13F and M13R primers (Supplementary Table S2).

### 4.5. Nucleotide Sequence Analysis and Comparison

The genomes of AcSBV-TW and AmSBV-TW were annotated by using NCBI ORFfinder, and proteins were predicted by using NCBI BLASTp [51]. The nucleotide sequences and the amino acid sequences of these two viruses were further compared to each other or to those of other SBVs from other countries.

For the nucleotide sequences, the whole genome sequence, 5′ UTR, ORF region, and 3′ UTR were compared; for the amino acid sequences, the polyproteins, structural proteins (rhv_like_1 and rhv_like_2), and nonstructural proteins (helicase, protease, and RNA-dependent RNA polymerase) of AcSBV-TW and AmSBV-TW were compared with other SBV sequence data from NCBI databases [52] (Table 1). Besides, the partial VP1 sequence of 3 AmSBV and 4 AcSBV from Taiwan were further compared to those of AmSBV-TW, AcSBV-TW, and other 54 SBVs. Multiple alignments of the sequences were obtained using ClustalX and edited in GeneDoc.

### 4.6. Phylogenetic Analysis

Phylogenetic analysis was performed based on the polyprotein sequences and partial VP1 region of the SBVs as follows. For the polyprotein phylogenetic analysis, the sequences of 54 SBV strains were obtained from the GenBank database and aligned, compared with AcSBV-TW and AmSBV-TW by using ClustalX and GeneDoc. For the partial VP1 phylogenetic analysis, 3 AmSBV and 4 AcSBV from Taiwan were included. Molecular Evolutionary Genetics Analysis Version 7.0 (MEGA7) was used for phylogenetic analyses of these two conserved domains with the neighbor-joining method. The nodes were determined via bootstrap analysis with 1000 replicates [53].

## 5. Conclusions

The whole genomes of SBV strains from *A. mellifera* and *A. cerana* were determined, and the origin of AcSBV-TW was indicated to be close to China, while AmSBV-TW presented novel genomic features. The cross-infection of *A. mellifera* with AcSBV was demonstrated in the apiaries, where *A. mellifera* and *A. cerana* were crossbreeding in Northern Taiwan in our previous report [33], suggesting that the variations identified in the genomes of AcSBV-TW and AmSBV-TW. According to the whole genome data, the sequences of 5′ and 3′ UTR revealed divergence compared to the polyprotein coding sequences either between AcSBV-TW and AmSBV-TW or among those of SBV from other countries, assuming there is less evolutionary pressure on the untranslated regions of the viral genomes. A comparison of partial VP1 region in Taiwan SBVs and phylogenetic analysis showed a deletion feature in VP1 region. The deletion feature in VP1 region, also mainly observed in most of AcSBV in other regions, suggested the host-preference phenomenon. However, it should be noted that some AmSBV also have a deletion in the VP1 region, it might be a consequence of cross-infection and viral–host adaptions. Therefore, cross-infection might be a high-risk factor for SBV resurgence [18,30,37,40]. For long-term surveillance, the features of VP1 in the genome sequences of SBV strains might provide molecular markers for the detection of SBV adaption in different honeybee hosts. More detailed investigations of this issue will be needed in the future.

## Figures and Tables

**Table 1:** Information on the sacbrood virus (SBV) strains used in this study.

No.	Name	Host	Total Size (bp)	Location	Accession No.	Reference
1	AmSBV-TW	*Apis mellifera*	8885	Taiwan	MN082651	This study
2	AcSBV-TW	*Apis cerana*	8776	Taiwan	MN082652	This study
3	AcSBV-IndTN-1	*Apis cerana*	8740	India	KX663835.1	[37]
4	AmSBV-Kor21	*Apis mellifera*	8855	Korea	JQ390591.1	[24]
5	AmSBV-Kor19	*Apis mellifera*	8784	Korea	JQ390592.1	[24]
6	South Australia_1	*Apis mellifera*	8821	Australia	KY887697.1	[38]
7	South Australia_2	*Apis mellifera*	8831	Australia	KY887698.1	[38]
8	South Australia_3	*Apis mellifera*	8848	Australia	KY887699.1	[38]
9	SBV-UK	*Apis mellifera*	8832	UK	NC_002066.1	[6]
10	AcSBV-Viet-LDst	*Apis cerana*	8832	Viet Nam	KJ959613.1	-
11	CSBV-SXYL-2015	*Apis cerana* (bee larvae)	8776	China	KU574662.1	[39]
12	Korean	*Apis cerana*	8792	Korea	HQ322114.1	-
13	CSBV-BJ	*Apis cerana* (bee larvae)	8857	China	KF960044.1	-
14	MD1	*Apis mellifera*	8861	USA	MG545286.1	[40]
15	MD2	*Apis mellifera*	8861	USA	MG545287.1	[40]
16	AmCSBV-SDLY	*Apis mellifera*	8794	China	MG733283.1	[40]
17	CSBV-LNQY-2009	*Apis cerana* (bee larvae)	8863	China	HM237361.1	[39]
18	CSBV-JLCBS-2014	*Apis cerana* (bee larvae)	8794	China	KU574661.1	[39]
19	AcSBV-Viet-SBM2	*Apis cerana*	8854	Viet Nam	KC007374.1	[41]
20	AcSBV-India-II10	*Apis cerana*	8550	India	JX194121.1	-
21	AcSBV-India-II2	*Apis cerana*	8680	India	JX270795.1	-
22	AcSBV-India-K1A	*Apis cerana*	8743	India	JX270796.1	-
23	AcSBV-India-K5B	*Apis cerana*	8700	India	JX270797.1	-
24	AcSBV-India-K3A	*Apis cerana*	8756	India	JX270798.1	-
25	AcSBV-India-S2	*Apis cerana*	8741	India	JX270799.1	-
26	AcSBV-India-II9	*Apis cerana*	8740	India	JX270800.1	-
27	AmSBV-Kor1	*Apis mellifera*	8837	Korea	KP296800.1	[36]
28	AmSBV-Kor2	*Apis mellifera*	8834	Korea	KP296801.1	[36]
29	AcSBV-Kor3	*Apis cerana*	8787	Korea	KP296802.1	[36]
30	AcSBV-Kor4	*Apis cerana*	8786	Korea	KP296803.1	[36]
31	AcSBV-Viet1	*Apis cerana*	8787	Viet Nam	KM884990.1	[30]
32	AcSBV-Viet2	*Apis cerana*	8786	Viet Nam	KM884991.1	[30]
33	AcSBV-Viet3	*Apis cerana*	8787	Viet Nam	KM884992.1	[30]
34	AmSBV-Viet4	*Apis mellifera*	8787	Viet Nam	KM884993.1	[30]
35	AcSBV-Viet5	*Apis cerana*	8784	Viet Nam	KM884994.1	[30]
36	AmSBV-Viet6	*Apis mellifera*	8836	Viet Nam	KM884995.1	[30]
37	VN3	*Apis mellifera*	8820	Australia	KY465673.1	[42]
38	VN2	*Apis mellifera*	8832	Australia	KY465674.1	[42]
39	VN1	*Apis mellifera*	8835	Australia	KY465675.1	[42]
40	QLD	*Apis mellifera*	8835	Australia	KY465678.1	[42]
41	SA	*Apis mellifera*	8823	Australia	KY465677.1	[42]
42	WA2	*Apis mellifera*	8832	Australia	KY465671.1	[42]
43	WA1	*Apis mellifera*	8832	Australia	KY465672.1	[42]
44	NT	*Apis mellifera*	8830	Australia	KY465679.1	[42]
45	TAS	*Apis mellifera*	8835	Australia	KY465676.1	[42]
46	AcSBV-Viet-BP	*Apis cerana*	8831	Viet Nam	KX668139.1	-
47	AcSBV-Viet-NA	*Apis cerana*	8791	Viet Nam	KX668140.1	-
48	AcSBV-Viet-BG	*Apis cerana*	8784	Viet Nam	KX668141.1	-
49	CSBV-SXnor1	*Apis cerana*	8705	China	KJ000692.1	-
50	CSBV-FZ	*Apis cerana*	8800	China	KM495267.1	[43]
51	CSBV-GZ	*Apis cerana* (bee larvae)	8740	China	AF469603.1	[5]
52	SBV-Brno	*Apis mellifera*	8832	Czech Republic	KY273489.1	-
53	SBV-Hynor	*Apis cerana* (bee larvae)	8779	Viet Nam	KJ959614.1	-
54	SBV-Sydney	*Apis mellifera*	8833	Australia	MF623170.1	-
55	SBV_MS	*Apis mellifera*	8828	Sweden	MH267698.1	-
56	SBV_MR	*Apis mellifera*	8830	Sweden	MH267697.1	-

-: unpublished.

**Table 2:** Comparison of genomic sequences and protein regions of AcSBV and AmSBV in Taiwan.

Genomic Region/Protein Region	Region Name	AmSBV-TW	AcSBV-TW	Identity (%)
Genomic region (nt)	Full length	8885	8776	90%
	5′ UTR	211	174	68%
	ORF region	8580	8526	90%
	3′ UTR	96	75	73%
Protein region (aa)	Polyprotein	2859	2841	95%
	rhv_like_1	176	176	95%
	rhv_like_2	188	188	97%
	Helicase	110	110	99%
	Protease	166	215	75%
	RdRp	292	292	96%

**Table 3:** Comparison of the nucleotide sequence homology (%) of different genomic regions of AcSBV-TW, AmSBV-TW, and 54 other SBV strains.

SBV Strains	Full Length (nt)		5′ UTR		ORF Region		3′ UTR	
	AcSBV-TW	AmSBV-TW	AcSBV-TW	AmSBV-TW	AcSBV-TW	AmSBV-TW	AcSBV-TW	AmSBV-TW
AcSBV-TW	-	90	-	68	-	90	-	70
AmSBV-TW	90	-	68	-	90	-	70	-
VN3	89	90	85	72	89	90	88	77
SBV-Sydney	89	90	84	71	88	90	84	71
VN1	89	90	85	72	89	90	88	77
AmSBV-Kor21	89	90	86	73	89	90	71	90
VN2	89	90	86	73	89	91	89	78
South Australia_1	88	90	87	71	88	90	76	63
South Australia_3	88	90	79	76	88	90	85	71
South Australia_2	88	89	87	70	89	90	85	71
QLD	89	90	86	72	89	90	89	78
AmSBV-Kor1	89	90	85	72	89	90	87	79
SA	89	90	85	72	89	90	89	78
WA2	89	90	87	73	89	90	89	78
WA1	89	90	86	72	89	91	89	78
NT	89	91	85	72	89	91	85	72
TAS	89	90	87	73	89	90	88	77
SBV_MS	89	90	83	75	89	90	77	64
SBV_MR	88	90	84	75	89	90	80	66
MD1	88	90	82	77	89	90	75	94
MD2	88	90	82	78	89	90	75	93
SBV-Brno	89	90	86	72	89	91	87	77
SBV-UK	89	90	84	70	89	90	87	77
AcSBV-Viet1	96	90	92	71	96	90	93	73
AcSBV-Viet2	96	90	89	69	96	90	93	73
AcSBV-Viet3	95	90	93	72	96	90	92	73
AmSBV-Viet4	95	90	89	71	95	90	92	73
SBV-Hynor	95	90	89	71	95	90	85	66
AcSBV-Viet5	95	90	88	69	96	90	85	70
AmCSBV-SDLY	96	90	88	77	96	90	94	70
CSBV-JLCBS-2014	95	90	84	76	95	91	88	76
AmSBV-Kor2	93	91	81	72	93	91	91	75
Korean	93	90	90	78	94	91	88	76
AcSBV-Kor4	94	90	84	70	94	91	90	75
AcSBV-Kor3	93	90	84	71	93	91	90	75
AmSBV-Kor19	93	90	86	73	93	91	88	76
CSBV-LNQY-2009	91	90	61	55	92	91	50	58
CSBV-FZ	96	91	91	72	97	91	92	73
CSBV-GZ	93	90	65	50	94	92	23	20
AcSBV-India-II10	88	87	-	-	91	91	-	-
AcSBV-India-II2	90	89	47	36	91	91	27	22
AcSBV-India-K1A	90	90	66	52	91	91	39	32
AcSBV-India-K5B	90	90	65	51	91	91	11	10
AcSBV-IndTN-1	90	90	68	53	91	91	47	37
AcSBV-India-K3A	90	90	61	47	91	91	50	41
AcSBV-India-S2	89	89	65	51	90	91	23	20
AcSBV-India-II9	90	90	67	52	91	91	50	41
AcSBV-Viet-BP	90	90	81	70	91	91	78	65
AmSBV-Viet6	91	91	85	71	91	91	84	76
AcSBV-Viet-LDst	91	91	83	72	91	91	82	68
AcSBV-Viet-NA	93	90	89	71	94	91	77	70
AcSBV-Viet-BG	92	90	83	70	93	90	66	54
AcSBV-Viet-SBM2	92	92	84	72	92	92	70	88
CSBV-BJ	91	91	82	69	91	91	68	87
CSBV-SXnor1	90	89	42	30	92	91	27	23
CSBV-SXYL-2015	91	90	81	68	91	91	23	20

-: Non comparable.

**Table 4:** Amino acid sequence homology (%) of AcSBV-TW, AmSBV-TW, and 54 other SBV strains.

SBVs	Polyprotein		rhv_like_1		rhv_like_2		Helicase		Protease		RdRp	
	AcSBV-TW	AmSBV-TW	AcSBV-TW	AmSBV-TW	AcSBV-TW	AmSBV-TW	AcSBV-TW	AmSBV-TW	AcSBV-TW	AmSBV-TW	AcSBV-TW	AmSBV-TW
AcSBV-TW	-	95	-	95	-	97	-	99	-	75	-	96
AmSBV-TW	95	-	95	-	97	-	99	-	75	-	96	-
VN3	94	97	96	97	96	98	99	100	88	85	96	96
SBV-Sydney	94	97	95	96	96	98	99	100	88	85	97	97
VN1	94	97	96	97	96	98	60	60	88	85	96	96
AmSBV-Kor21	94	97	90	91	96	98	98	99	88	85	97	97
VN2	94	97	96	97	96	98	99	100	88	85	97	97
South Australia_1	94	97	95	96	96	98	99	100	88	85	96	96
South Australia_3	94	97	95	96	96	98	99	100	88	85	97	97
South Australia_2	94	97	95	96	96	98	99	100	88	85	97	97
QLD	94	97	96	97	96	98	99	100	87	83	97	97
AmSBV-Kor1	94	97	96	97	96	99	99	100	88	84	96	96
SA	94	97	96	97	96	98	99	100	89	83	97	97
WA2	94	97	96	97	96	98	99	100	88	85	96	96
WA1	94	97	96	97	96	98	99	100	88	84	97	96
NT	95	97	96	98	96	98	99	100	98	77	97	97
TAS	94	97	95	96	96	98	99	100	88	85	97	96
SBV_MS	94	97	96	97	96	98	99	99	88	85	96	96
SBV_MR	94	97	95	96	96	98	99	99	88	85	96	96
MD1	95	97	96	97	96	98	99	99	84	88	97	97
MD2	95	97	96	97	96	98	99	99	88	85	97	97
SBV-Brno	95	97	96	97	96	98	98	99	98	77	97	97
SBV-UK	95	98	96	97	96	98	99	100	98	77	97	97
AcSBV-Viet1	98	96	92	91	98	96	100	99	76	98	97	96
AcSBV-Viet2	98	96	92	91	98	96	100	99	76	98	97	96
AcSBV-Viet3	97	95	92	91	98	96	99	98	98	75	97	96
AmSBV-Viet4	97	96	94	95	98	96	100	99	99	75	97	96
SBV-Hynor	97	95	92	91	99	96	100	99	96	75	93	92
AcSBV-Viet5	97	95	92	90	99	96	100	99	76	98	93	92
AmCSBV-SDLY	97	95	92	91	100	97	100	99	76	98	97	96
CSBV-JLCBS-2014	97	95	94	95	100	97	100	99	91	83	97	96
AmSBV-Kor2	96	96	95	97	97	96	100	99	88	85	93	93
Korean	96	96	95	97	97	96	100	99	88	85	97	97
AcSBV-Kor4	97	96	94	96	97	96	99	98	88	84	94	92
AcSBV-Kor3	96	96	94	96	97	96	99	98	88	84	94	94
AmSBV-Kor19	96	96	94	95	97	96	100	99	88	85	96	96
CSBV-LNQY-2009	94	95	94	96	96	97	98	99	79	73	97	96
CSBV-FZ	97	95	98	97	99	96	100	99	67	86	98	97
CSBV-GZ	95	96	94	97	97	97	99	98	79	75	98	98
AcSBV-India-II10	93	94	84	84	96	98	99	100	96	75	96	97
AcSBV-India-II2	94	95	93	93	95	96	99	100	96	75	96	96
AcSBV-India-K1A	93	95	93	93	96	98	99	100	97	76	96	96
AcSBV-India-K5B	94	95	93	91	97	97	99	100	97	75	96	96
AcSBV-IndTN-1	94	95	92	93	96	97	99	100	97	76	95	95
AcSBV-India-K3A	94	96	90	90	96	96	99	100	98	76	93	94
AcSBV-India-S2	93	95	90	90	97	97	97	98	91	70	89	88
AcSBV-India-II9	94	95	92	93	96	97	99	100	97	76	95	96
AcSBV-Viet-BP	93	95	82	82	94	95	96	97	95	74	94	95
AmSBV-Viet6	94	96	81	81	95	95	99	100	97	75	96	97
AcSBV-Viet-LDst	94	96	84	84	95	95	99	100	97	75	96	97
AcSBV-Viet-NA	96	96	92	91	95	94	99	100	96	75	93	93
AcSBV-Viet-BG	95	95	85	85	100	97	99	100	96	75	94	95
AcSBV-Viet-SBM2	95	97	95	96	97	97	99	100	73	97	96	97
CSBV-BJ	94	95	81	82	96	96	98	99	78	75	96	96
CSBV-SXnor1	94	95	95	96	96	97	98	99	78	75	97	96
CSBV-SXYL-2015	94	95	94	95	97	97	96	97	87	85	96	96

-: Non comparable.

## Data Availability

The sequences generated in this study were submitted to NCBI GenBank and also are available from the corresponding author (Yu-Shin Nai) on reasonable request.

## Supplementary Materials

The following are available online at https://www.mdpi.com/2076-0817/10/1/14/s1, Supplementary Table S1: Information of sampling sites; Supplementary Table S2: Primers used in this study.
